# Micromanagement During Clinical Supervision: Solutions to the Challenges

**DOI:** 10.7759/cureus.23523

**Published:** 2022-03-26

**Authors:** Anuradha Mookerjee, Becky Li, Bhawana Arora, Rakesh Surapaneni, Vijay Rajput, Monica Van de Ridder

**Affiliations:** 1 Internal Medicine, Cooper Medical School of Rowan University, Camden, USA; 2 School of Medicine, Nova Southeastern University Dr. Kiran C Patel College of Allopathic Medicine, Fort Lauderdale, USA; 3 Pediatric Emergency Medicine, Spectrum Health Medical Group, Grand Rapids, USA; 4 Oncology, Baylor Scott & White Health, Round Rock, USA; 5 Medical Education, Nova Southeastern University Dr. Kiran C. Patel College of Allopathic Medicine, Fort Lauderdale, USA; 6 College of Human Medicine, Michigan State University, Grand Rapids, USA

**Keywords:** micromanagement, medical resident education, learner autonomy, clinical learning environment, clinical supervision

## Abstract

Learner autonomy is an invaluable asset in graduate medical education, preparing the trainee to independently face challenges in the future professional settings. Educational institutions face the difficult task of providing a balance between learner autonomy and supervision. In graduate medical education, trainees often prefer less supervision than what is imparted by their attending physician. This increased supervision comes at the cost of learner autonomy and has not exhibited improvement in patient outcomes or safety. When attendings exhibit control over details, the trainees may label them as “micromanagers”. Cardinal features of a micromanager include excessively requesting updates, insisting that the task be done their way, and scrutinizing every detail. This micromanaging behavior is non-conducive to the learning environment and may even contribute to supervisor burnout. The business literature reveals a debate about this very topic. Unfortunately, there is still a lack of literature on micromanagement in graduate medical education.

Although a conglomerate of internal factors may lead to excessive supervision in an academic medical institution, we surmise that micromanagement exists because of a complex dynamic between three drivers: accountability, trust, and autonomy. When trainees are held accountable, they learn to take ownership for their actions which leads to establishment of trust which further enables motivation and gaining of autonomy. Supervising attendings should ideally be able to comfortably adjust their level of supervision based on their trust and the trainee’s competence, accountability, and autonomy. The micromanaging physician is unable to do so, and this can have a detrimental effect on the learner.

Micromanagement can be perceived by some as a beneficial component during the early immersion of the trainee with the rationalization for better patient outcomes and safety. However, in the long term, it threatens the learning environment and erodes the complex relationship between accountability, trust, and autonomy. We recommend an action plan to mitigate micromanagement at three levels-the micromanager, the micromanaged, and the organizational structure-and hope that these solutions enhance the learning environment for both the trainee and supervisor.

## Introduction and background

During clinical training, novice learners adapt to navigating the complex working environment while developing their knowledge, skills, and attitude. The Accreditation Council for Graduate Medical Education (ACGME) in the United States provides the guidelines for varying levels of supervision a resident physician receives from the attending physician, starting from direct supervision and transitioning gradually to indirect supervision [1]. With an appropriate level of supervision, trainees provide high-quality patient care, progressively earning autonomy and trust of faculty before graduating to become independent practicing physicians [1].

The appropriate level of supervision a learner should receive is up to the subjective interpretation of the learner and/or supervisor in clinical care. When surveying residents and attendings, residents tend to prefer less supervision than the amount their supervising attending physician wishes to give [2,3]. If the supervision reaches an excessive level, attendings can be known amongst the residents as “micromanagers” [4]. Micromanagement is defined as a supervisory style of “hovering” and directly commanding all of the details, rather than giving space to the trainee assigned to the task [5]. In the context of attending-resident supervision, micromanaging attendings tend to scrutinize the decisions already made appropriately by the trainees. Some examples of micromanaging behaviors may include things such as delaying patient discharge, disputing over medications, and requesting unnecessary consultations (Table 1) [4]. This increased supervision/scrutiny has been shown not to have any reduction in medical errors and instead interferes with resident education [6]. As a result, micromanagement has been perceived by residents to impede their autonomy in the clinical decision making process thereby affecting their learning and professional development [7]. This article was previously presented as an oral presentation at the 2021 Group of Educational Affairs (GEA) Joint Regional Conference on April 20-22, 2021 and the 2021 Australian & New Zealand Association for Health Profession Educator Festival Program on July 8, 2021. It was presented as a poster at the 2021 Association for Medical Education in Europe Conference on August 29, 2021.

**Table 1:** Micromanaging behavior examples in a healthcare environment H&P: history and physical

Micromanaging behaviors	Examples in the healthcare environment
Scrutinizing every detail	Example 1: “The cardiologist who demanded to know each patient’s furosemide dose”[4]
Excessively requesting updates	Example 1: The in-house hospitalist asks for frequent updates on every patient, including non-critical patients. Example 2: When the attending physician demands to be included in a group conversation involving the junior and senior residents.
Insists tasks are done micromanagers’ way or else frustrated	Example 1: The attending tells the resident, “I told you that I wanted imaging prior to ordering laboratory testing (when there is not a clear evidence-based approach).” Example 2: A resident wrote the medical note (H&P) in bulleted format, and the attending is frustrated with this writing style.
Unsatisfied with others’ results	Example 1: An attending surgeon gets upset at an intern for the patient’s infected suture and blaming the intern. Example 2: The gastroenterologist blames the trainee assisting in colonoscopy for missing a polyp.
Delaying task completion	Example 1: An attending delays patient discharge by requesting unnecessary consultations without appropriate justification. Example 2: “The oncologist who kept a patient hospitalized to receive outpatient chemotherapy”[4]
Disallow learner decision-making autonomy	Example 1: The attending physician does not engage the trainee in decision making process to come up with the treatment plan. Example 2: The attending insists on the trainee calling several consultants on a case, rather than first asking the trainee “what do you think we should do?”
Disputes over details	Example 1: The attending does not give an evidence-based reason for antibiotic preference when there is an equally appropriate alternative according to guidelines.
Taking pride in correcting others’ mistakes	Example 1: The attending tells the resident, “I am the one who changed the medication to the right one. If I were not here, you would have made an error.” Example 2: The attending tells the resident, “It is good that you consulted me. You did not look at the patient’s feet. That is where you went wrong.”

## Review

Factors promoting micromanaging behaviors

**Figure 1:**
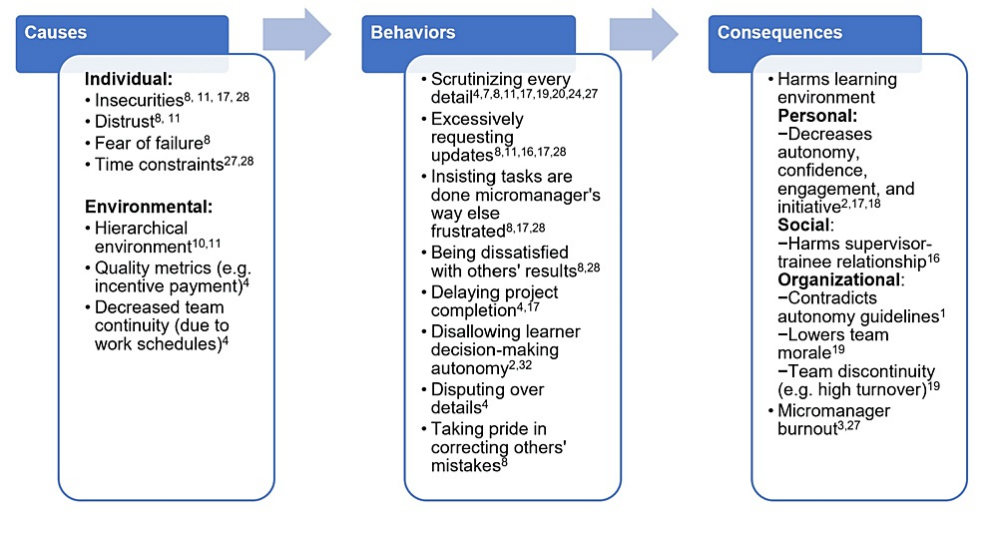
Causes of micromanagement, micromanaging behaviors, and their consequences Source: [1-4,7,8,10,11,16-20,24,27,28,32]

To understand micromanaging behaviors, it is important to understand the individual and organizational environment factors that allow such behaviors to appear in the academic healthcare center (Figure 1). On an individual level, micromanagers may have insecurities, distrust, fear of failure, or even time constraints, spurring them to dictate in an authoritarian manner [8,9]. In regards to the work environment, micromanagement thrives in hierarchical working environments because of the strict boundaries placed on people [10,11]. A healthcare team can be an example of such hierarchical environments, with the attending at the top of the pyramid [12]. Certain other factors that promote micromanagement are the present-day quality metrics and increase transition of care [4]. Currently, quality metrics are now quantified and factored into incentive payment and accreditation. These quality metrics include "resident-level" decision-making, such as compliance with flu vaccine, thromboprophylaxis, and smoking cessation, which can explain why some attendings are more inclined to “hover” and closely monitor resident actions and management plans [4]. Implementation of resident duty-hour restrictions have correlated with a subsequent rise in 24/7 in-house hospitalists and multiple handoffs [13]. This shift work has fragmented team interactions and restricted the resident’s ability to manage the ongoing care of patients admitted during off-hours [4]. Thus, although duty-hour regulations have been created to improve resident well-being, residents have reported that these regulations have negative effect on their learning environment [14]. With the attending serving as the ultimate bearer of responsibility and safety net, current environment fosters the attending-resident relationship to be more of scrutiny rather than supervision.

Impact of micromanagement on the learning environment

We believe that micromanaging behaviors have a negative effect on the overall learning environment that nurture learner’s professional development. Micromanagement harms multiple components of the learning environment framework: personal, social, and organizational [15]. The personal component focuses on the individual learner’s growth, engagement, and autonomy [15]. Micromanagement threatens this component in all three aspects. The trainee is not able to have the freedom to form the plan of care or explore another comparable treatment because the micromanaging supervisor dictates every detail of patient care [16]. Learners can become apathetic and doubtful of their own knowledge and skills, leading to decreased motivation, engagement, and initiative [2,17,18]. The social component incorporates interactions in patient care, learning, and teaching [15]. The supervisor-trainee relationship becomes tainted because the micromanager’s actions convey that there is distrust of the trainee [16]. The organizational component is associated with ACGME accreditation rules, organizational culture/practices/policies, and curriculum resources [15]. Micromanaging attitudes and behaviors contradicts ACGME rules, which emphasizes the responsibility of the educational institution to “promote progressive autonomy” [1]. Micromanagement can also negatively affect organizational culture as stifled members of a healthcare team can have low morale which leads to a high turnover rate [19]. Although residents themselves may not have a turnover rate at their institution, this high turnover rate can still adversely affect them as they cannot benefit from the consistency and morale of their team members.

Micromanagement impairs autonomy necessary for trainee development

The ACGME highlights autonomy in residency training requirements because autonomy is what makes a resident become an independent and skilled physician [1,20]. Similarly, the American Association of Colleges of Nursing (AACN) values increasing autonomy through experiences that transition learners to become “competent and confident” practitioners [21]. Cognitive, physical, emotional, and cultural safety enable learners to be out of their comfort zone, which is necessary for effective learning. The right balance between learner autonomy and supervision is ideal for critical thinking and self-reflection [22]. When learners are given autonomy, they are motivated to be accountable for the patient, an attitude that is vital in effective clinical practice [23]. Studies of students given autonomy revealed an associated increase in intrinsic motivation, as well as a desire to learn and take on challenges [18]. Even if the competence is achieved, there is no motivation to continue unless the competence was perceived to be attained independently [18]. In contrast, examples of micromanaging behaviors such as dictating orders may decrease intrinsic motivation because trainees may not feel empowered with forming the final plan of care [18].

Of course, it is also important to add that the health professional trainees must be given autonomy within reasonable limits that would not compromise patient safety. Patient care should always be prioritized, and supervising attendings should “adjust their level of supervision”, depending on the level of urgency for critical action and trainee competence [20]. Therefore, residency programs ideally should have a balance between trust, accountability, and autonomy while still prioritizing patient care and safety [23].

Short-term benefits of micromanagement

Although micromanagement in excess is detrimental to the learning environment, there may be scenarios in which a higher level of supervision might be useful. The business literature cites examples such as inadequate results, customer complaints, or changes in leadership, employee, or organizational strategy when micromanagement might be applied by the supervisors to achieve optimum results [24,25]. Similarly, in the healthcare environment, direct and close supervision can also be applied during the initial stages of the trainee’s educational development. Sometimes attendings may feel the need to shift their supervisory style in order to control the outcome and guarantee patient safety in high-risk situations or with novice trainees [19,24]. To prevent being perceived as a “micromanager”, it is important for the faculty to communicate that the excessive level of supervision is not due to the distrust of the learner [24]. This communication should ideally be done prior to micromanaging actions in real-time, not retroactively. However, as mentioned previously, randomized controlled clinical trials have shown no change in patient outcomes and safety with increased excessive supervision [6,26].

Micromanaging preferably should only be used for short-term goals. In the long-term, micromanagement will ultimately restrict a team’s growth and morale. In the business world, it is one of the top reasons why employees resign [19]. Micromanagement may be a catalyst for a physician’s own burnout, which can happen when they expend a great deal of energy to oversee every minute detail [19,27].

Solutions for the micromanager, the micromanaged, and the organization

Below, we outline an action plan to address micromanagement at three levels (Table 2).

**Table 2:** Suggested solutions for the micromanager, micromanaged, and organization

Role	Action items	Tools
Micromanager	Identify the problem (self-awareness). Self-reflect (what is causing this behavior?). Shift supervisory style to one of delegation. Gather feedback through direct communication and through program leadership	Self-assessments and multisource feedback
Micromanaged	Share experiences with peers (as part of coping process). Report issues (share experiences with program leadership to better facilitate communication and brainstorm solutions). Earn entrustment (resident behaviors can involve self-management, relationships, self-advocacy, and patient-centeredness).	Educational sessions addressing micromanaging issues, conversations with peers, and surveys
Organization	Encompass a psychologically safe environment to reduce learner anxiety through educational workshops and blameless reporting. Create a culture of respect and safety in the organization.	Coaching (professional or peer-to-peer), faculty development workshops, and have annual evaluations of supervisory performance

The Micromanager (Supervising Faculty)

For micromanagers, the first step is to be self-aware of their behaviors [28]. There are self-assessments that faculty can take if they suspect they are becoming a micromanager [11,24]. There is also a self-assessment to determine overall leadership style [19]. In addition, attendings should encourage and accept regular feedback from the team and peers. This will increase self-awareness of the attendings’ supervising behavior and promote a culture of communication and acceptance of constructive criticism [19,29]. Attendings should also regularly meet with residency program leadership, who also gather feedback from residents and can bring up any issues. Once a micromanaging problem is identified, faculty can then self-reflect, seek support, and challenge their rationalizations in order to modify their micromanaging behaviors [19]. Common rationalizations for their behavior include patient safety, avoiding medical errors, and improving efficiency. For addressing complex reasons such as individual insecurities and fears, it will likely require additional time and external aid, such as coaching and/or cognitive behavioral therapy. In the circumstance that micromanaging faculty cannot self-identify behaviors, we suggest multi-source feedback, faculty development workshops, and coaching, also addressed in suggested solutions for the organization.

Following identification and self-reflection, micromanaging faculty should work towards achieving a balance between learner autonomy and clinical supervision. One method used in resident education is the “backstage” approach: attendings are aware of all decisions but the residents are given the autonomy to make those decisions [2]. This approach is used in independent rounds, in which residents conduct patient rounds separately from the attendings [22]. Independent rounding has been shown to increase trainee-perceived autonomy, critical thinking, and confidence in decision-making [22]. There is also a clinician teaching toolbox available detailing the steps and example phrases that an attending can use to nurture clinical autonomy [20]. The toolbox mentions that although trainees must prove that they are ready for entrustment, attendings should give opportunities to prove their readiness [20]. Probing residents for their decision-making reasoning will not only demonstrate their ability to approach a situation and synthesize information, but it will also exhibit their readiness to handle more autonomy [20].

Furthermore, supervising faculty should place efforts to shift their supervisory style towards delegation and becoming an adaptive attending [19,20,27]. Labeled as the “antonym” of micromanagement, delegation is defined as assigning work with a condition of mutual understanding between assigner and assignee of the results and methods [9,10]. Failure to delegate is believed to ultimately hinder professional growth by limiting the task to one person, diminishing the effectiveness of having a team [9]. The adaptive faculty also benefits from delegation by being able to focus on other duties, thus increasing team efficiency and effectiveness [9]. Delegation is an "investment of time" and a "calculated risk" for attendings as failure is a possibility [9]. Risk of failure is minimized by allotting a suitable amount of time to provide instruction [9]. The responsibility of completing the delegated task still lies with the attending; thus, it is in the attending's best interest to communicate instructions clearly and evaluate the situation appropriately [9]. Therefore, in the context of medical education, supervising faculty can address micromanagement by relinquishing control of activities (e.g. allowing residents to take lead in the rounds), delegating tasks (e.g. delegating teaching responsibilities to senior residents), and facilitating feedback (from learner to team members and faculty) [30]. A specific example is for attendings to pause and consider: can this wait until rounds the next day, or does this require a call to the resident currently at 11 pm?

**Figure 2:**
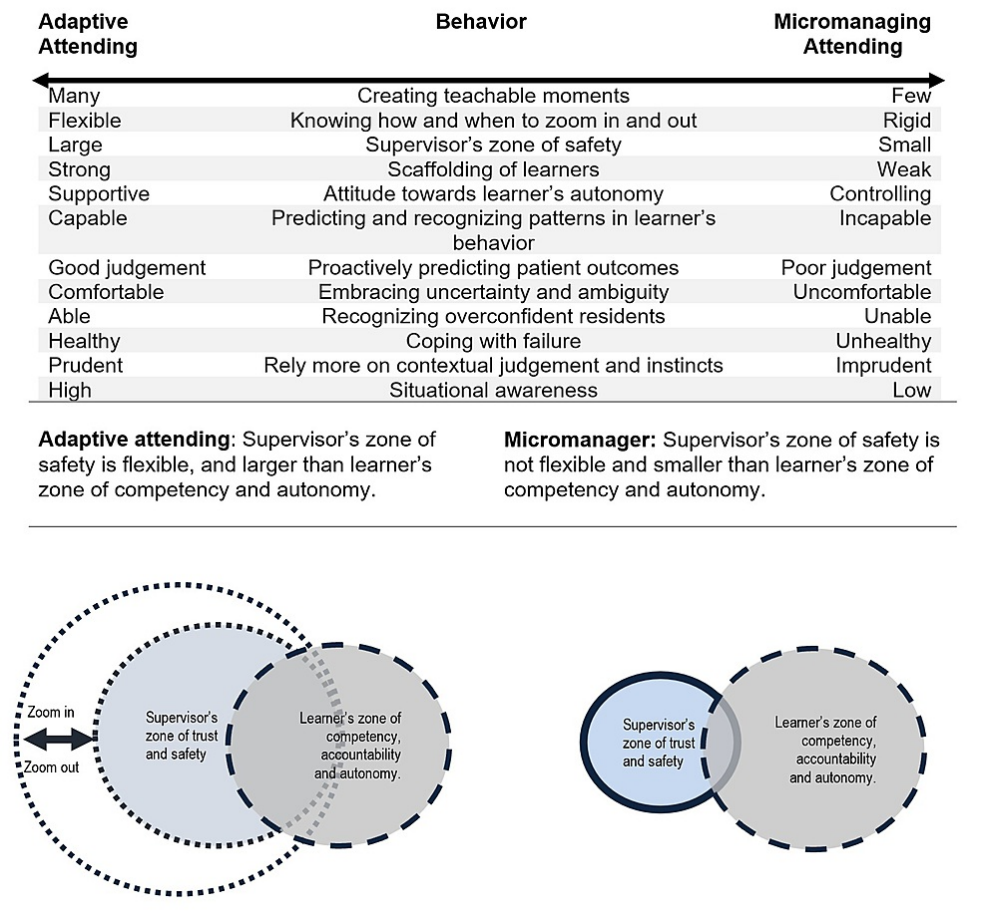
An adaptive attending versus a micromanager in behaviors and the relationship of learner’s zone of competency with the supervisor’s safety zone

In our model (Figure 2), we demonstrate that the micromanaging attending does not create a flexible “safety-zone” for the trainee. We refer the “safety-zone” as the area where trainees have psychological safety and can engage in independent decision making which comes with some “inherent risk” but can also request for help if needed [31]. The narrow and rigid safety-zone of the micromanager prevents the trainee from gaining opportunities for autonomy. This results in an inadequate safe space as well as a tense and restricted learning environment. Our model contrasts this micromanaging attending to the adaptive attending, who tailors the level of supervision based on the trainee’s zone of competency, accountability, and autonomy and therefore has a flexible safety-zone. In medicine, we need an adaptive and coach-like attending to provide safety-zones that can be “flexible” to fit the needs of trainees and the team (Figure 2).

The Micromanaged (Learner)

Involving residency program leadership is a great tool in facilitating any conflict between trainees and attendings. Attendings evaluate trainees, thus trainees may be apprehensive about raising concern about them. Trainees generally have regular check-in meetings with program leadership but should mention issues straightaway. Trainees who believe they are being micromanaged can work towards earning entrustment from their supervisor. A survey of attendings and residents revealed that attendings give autonomy based on their level of trust [32]. Trainee behaviors that can increase supervisors’ trust comprise of self-management, relationships, self-advocacy, and patient-centeredness [3]. For self-management, learners can foster their emotional intelligence, receptiveness to feedback, stress management, and confidence [3]. For trainees, relationships can be further developed with their supervisor, physician team members, interprofessional team members, and the healthcare system [3]. Strengthened peer relationships can also benefit micromanaged trainees because confiding with peers can help with their coping process. To address team disruption due to work scheduling, trainees can develop their ability to quickly adapt to new teams, also termed as “teaming” [33]. The trainees can self-advocate by showing interest or even explicitly advocating for entrustment [3]. They can illustrate patient-centeredness through their relationships with patients and families as well as their ownership of patient care [3]. In the business world, there are courses that the micromanaged can take in order to address their challenges [34-37]. We suggest that there be courses created for healthcare trainees as well.

The Organization

Earlier we emphasized the importance of attendings meeting regularly with residency program leadership to receive feedback to help attendings identify their own micromanaging behaviors. Similarly, their departmental leadership should perform their own evaluations and provide feedback periodically. Departmental leadership can also work together with attendings in providing individualized plans targeting their personal improvement.

The organization should cultivate an atmosphere of trust and autonomy. It has been shown that duty-hour restrictions can encourage micromanaging behaviors, which are perceived by the residents to have a negative effect on their learning environment. Another study revealed that over half of residents reported worsened continuity of care, and senior residents reported heavier workload [38]. The current fast pace of trainees’ work schedules prevents residents from building long-term relationships with patients and attending physicians. These relationships are essential for developing trust. We suggest identification of opportunities for team continuity, team accountability, and resident autonomy while adhering to the duty-hour guidelines. Specific examples include continuity-enhanced handovers and a culture of accepted responsibility from every single team member [39]. Organizations should instill the impression that all team members assume professional responsibility regardless of recency and experience (e.g. moonlighters, covering hospitalists, non-medical professionals) [39]. Individual programs should additionally consider if they would benefit from incorporating duty-hour flexibility (i.e. not requiring specified shift lengths or time off between shifts), which has been found to not compromise patient safety outcomes [40]. Allowing flexibility to duty-hour rules has shown to decrease residents’ negative perceptions on duty hours’ effect on autonomy and increased program director satisfaction regarding the learning environment [41]. However, this duty-hour flexibility trial was associated with lower resident satisfaction with educational quality, and thus still requires more exploration as to how to implement flexibility in a way that would improve resident learning [41].

In relation to promoting a culture of communicating and accepting constructive criticism, organizational structures should encompass psychological safety to allow team members to comfortably discuss issues such as micromanagement. Psychological safety is defined as the willingness to express oneself based on beliefs of how others will respond [42]. Having psychological safety reduces learner anxiety and allows for the development of positive interpersonal behaviors, such as seeking help, requesting feedback, and speaking up on medical errors-which are all conducive for the learning environment [42]. Creating a psychologically safe environment allows members of an organization to learn from failures [43]. This safe environment should also promote a culture of respect for all learners and organizational members to feel worthy and valued [44]. Examples of organizational adaptations for psychological safety involve “blameless reporting” and educational workshops [43]. Anonymous reporting of mistakes increases psychological safety because the reporter does not have to fear any negative consequences. There was an observed increased rate of reported failures when “blameless reporting” was implemented in the hospital setting, a result of increased psychological safety [43]. This “blameless reporting” is the first step to identifying issues such as micromanagement. The ACGME is responsible for creating residency program requirements, which do include residents having a system to report patient safety errors and allowing them to confidentially report/address unprofessional behavior [1]. However, it would be additionally effective for them to advocate for all programs to create psychologically safe environments through honest disclosures and blameless reporting. Finally, an organizational workshop on micromanagement would allow members to have increased awareness if they are being micromanaged or if they are becoming micromanagers. An alternative to educational workshops could be offering professional or peer-to-peer coaching.

## Conclusions

Micromanagement is a supervisory style that is not often addressed in healthcare. Micromanagement consists of excessive supervision and is detrimental to the learner, micromanager, and overall organization. It can lead to impaired trainee autonomy, which can further impair their smooth transition into independent practitioners. It can also have negative effects on the learning environment such as fragmented supervisor-learner relationships and pernicious organizational culture. The causes for micromanagement are multifactorial and should be addressed on multiple levels, from the micromanaging supervisor to the organizational structure. We recommend that the supervising attendings should be able to tailor their level of supervision to the learner’s zone of competency, accountability, and autonomy. We also suggest micromanagement be explored in future healthcare literature to increase awareness and discover novel solutions.
